# Treatment and survival of rectal cancer patients over the age of 80 years: a EURECCA international comparison

**DOI:** 10.1038/s41416-018-0215-6

**Published:** 2018-07-30

**Authors:** Yvette H. M. Claassen, Nina C. A. Vermeer, Lene H. Iversen, Elizabeth van Eycken, Marianne G. Guren, Pawel Mroczkowski, Anna Martling, Antonio Codina Cazador, Robert Johansson, Tamara Vandendael, Arne Wibe, Bjorn Moller, Hans Lippert, Harm J. T. Rutten, Johanneke E. A. Portielje, Gerrit J. Liefers, Fabian A. Holman, Cornelis J. H. van de Velde, Esther Bastiaannet

**Affiliations:** 1 Department of Surgery, Leiden University Medical Center, Leiden, The Netherlands; 2 Department of Surgery, Aarhus University Hospital, Aarhus, Denmark; 3 Danish Colorectal Cancer Group (DCCG.dk), Copenhagen, Denmark; 4 Belgian Cancer Registry, Brussels, Belgium; 5 Department of Oncology and K.G. Jebsen Colorectal Cancer Research Centre, Oslo University Hospital, Oslo, Norway; 6 Institute for Quality Assurance in Operative Medicine Ltd at Otto-von-Guericke University Magdeburg, Magdeburg, Germany; 7 Department for General and Visceral Surgery, Elisabeth Hospital, Kassel, Germany; 8 Department of Molecular Medicine and Surgery, Karolinska Institutet, Stockholm, Sweden; 9 Department of Surgery, Generalitat de Catalunya, Barcelona, Spain; 10 Department of Radiation Science, Oncology, Umeå University, Umeå, Sweden; 11 Institute of Cancer Research and Molecular Medicine, Norwegian University of Science and Technology—NTNU, Trondheim, Norway; 12 Cancer Registry of Norway, Oslo, Norway; 13 Department of Surgery, Catharina Hospital, Eindhoven, The Netherlands; 14 GROW: School of Oncology and Developmental Biology, University of Maastricht, Maastricht, The Netherlands; 15 Department of Gerontology & Geriatrics, Leiden University Medical Center, Leiden, The Netherlands

**Keywords:** Rectal cancer, Epidemiology

## Abstract

**Background:**

The optimal treatment strategy for older rectal cancer patients remains unclear. The current study aimed to compare treatment and survival of rectal cancer patients aged 80+.

**Methods:**

Patients of ≥80 years diagnosed with rectal cancer between 2001 and 2010 were included. Population-based cohorts from Belgium (BE), Denmark (DK), the Netherlands (NL), Norway (NO) and Sweden (SE) were compared side by side for neighbouring countries on treatment strategy and 5-year relative survival (RS), adjusted for sex and age. Analyses were performed separately for stage I–III patients and stage IV patients.

**Results:**

Overall, 19 634 rectal cancer patients were included. For stage I–III patients, 5-year RS varied from 61.7% in BE to 72.3% in SE. Proportion of preoperative radiotherapy ranged between 7.9% in NO and 28.9% in SE. For stage IV patients, 5-year RS differed from 2.8% in NL to 5.6% in BE. Rate of patients undergoing surgery varied from 22.2% in DK to 40.8% in NO.

**Conclusions:**

Substantial variation was observed in the 5-year relative survival between European countries for rectal cancer patients aged 80+, next to a wide variation in treatment, especially in the use of preoperative radiotherapy in stage I–III patients and in the rate of patients undergoing surgery in stage IV patients.

## Introduction

Colorectal cancer is the second most common cancer in Europe and is the second cause of death from cancer, with an estimated number of 215 000 deaths in 2012 in Europe.^1^ Rectal cancer is predominantly a disease of older patients, as the median age at diagnosis is 69 years.^2^ With the ageing population the number of older rectal cancer patients is expected to increase further. Older patients often have more comorbidities, an increased complication rate and a poorer prognosis.^3^ The evidence regarding the most optimal treatment for older rectal cancer patients is rather limited, because older patients are frequently excluded from randomised clinical trials.

Surgery is the cornerstone in the curative treatment of rectal cancer. The outcome of rectal cancer has improved dramatically after the introduction of total mesorectal excision (TME) surgery, the recognition and evaluation of the circumferential resection margin and after the introduction of neoadjuvant chemoradiotherapy.^4–7^ Although treatment guidelines vary between countries, most agree that patients with stage I disease (T1-2N0M0) should undergo surgery without neoadjuvant therapy, and that patients with locoregional advanced disease stages need neoadjuvant chemoradiotherapy. Most countries apply preoperative radiotherapy or chemoradiotherapy for defined subgroups of patients. However, unresolved questions remain about the fractionation and duration of radiotherapy (short course vs. long course), optimal time to surgery and the benefit of the addition of chemotherapy. In general, when downsizing of the tumour is desired, treatment with chemoradiotherapy and delayed surgery is preferred, and for less-advanced tumours short-course radiotherapy can be used.^6,8^ For older or frail patients, short-course radiotherapy with delayed surgery may be preferred over long-course chemoradiotherapy, and also dose reduction or omitting the chemoradiotherapy could be considered.^9,10^

For rectal cancer patients with limited metastatic disease (stage IV), a treatment strategy with curative intention may combine a R0-resection of the primary tumour as well as resection of the metastases, often after induction treatment.^11^ However, most stage IV patients are incurable and palliative chemotherapy (with or without targeted agents) is the therapy of choice, although some patients are not eligible for chemotherapy due to frailty or comorbidity.^11^

Comparative effectiveness research has gained interest over the years.^12,13^ Given that randomised clinical trials are not feasible for older patients and that outcomes should reflect a real-world clinical scenario, comparative effectiveness research on population-based observational data is a very suitable way to gain new insights in the best treatment strategies in geriatric oncology. Therefore, population-based data of rectal cancer patients aged 80+ of five different European countries (Belgium (BE), Denmark (DK), the Netherlands (NL), Norway (NO) and Sweden (SE)) were collected. The current study aimed to compare treatment strategy and survival in rectal cancer patients in these five European countries, separately analysed for patients with stage I–III and stage IV disease.

## Materials and methods

### Data and study population

Patients diagnosed with rectal cancer between January 2001 and December 2010 from BE, DK, NL, NO and SE, of 80 years of age or older were included. Data were obtained from the Belgian Cancer Registry, the Danish Colorectal Cancer Group database,^14^ the Netherlands Cancer Registry, the Norwegian Cancer Registry supplemented with data from the Norwegian Colorectal Cancer Registry^15^ and the Swedish Colorectal Cancer Registry. For BE, only data of the period 2004–2010 were available. All rectal cancer patients, defined as DC20 of the International Classification of Diseases and Related Health Problems, with all stages of disease were included.^16^ The TNM Classification of Malignant Tumours (fifth, sixth or seventh edition) was used for defining tumour stage.^17^ Tumour stage was based on pathological stage; in cases where this was missing clinical stage was used. Pathological stage consisted of ypTN stage (patients who received radiotherapy or chemoradiotherapy following delayed surgery) or pTN stage (patients receiving immediate surgery). In the current study patients were divided into stage I–III and stage IV disease. In addition to survival data, data collection consisted of the variables surgery (yes/no), preoperative radiotherapy (yes/no), chemoradiotherapy (yes/no), adjuvant chemotherapy (yes/no), radiotherapy without surgery (yes/no) and preoperative chemotherapy (yes/no). Surgery was defined as surgical resection of the tumour, irrespective of curative or palliative intent. Local excisions were included while construction of a stoma without tumour resection and endoscopic techniques were excluded. Follow-up time was defined as the time from date of diagnosis until death or until end of follow-up (censored). In case of missing follow-up data, patients were excluded from survival analyses.

### Statistical analyses

Relative survival (RS), expressed as relative excess risk (RER) and adjusted RER (adjusted for sex, stage and age), and corresponding 95% confidence interval (CI) were calculated for each country.^18,19^ RS was defined as the ratio of the survival observed in the cohort and the expected survival based on the matched general population in the respective countries. National life tables of the respective countries were used to estimate expected survival. RER and 95% CI were calculated for the differences between countries, using a multivariable generalised linear model with a Poisson distribution, based on collapsed RS data, using exact survival times. RS and RER were truncated at 5 years.

Analyses were performed separately for patients with stage I–III disease and patients with stage IV disease. In case of missing stage, patients were excluded from the stratified analyses. Treatment strategy and RER were compared between neighbouring countries: DK vs. SE, NO vs. SE and BE vs. NL.

STATA/SE version 12.0 was used for all analyses. A *p*-value < 0.05 was considered as statistically significant.

## Results

### Patient characteristics, tumour characteristics and median follow-up

In total 19 634 rectal cancer patients were included (Table 1). The majority of patients were between 80 and 84 years in all countries. In DK, 26.2% (*n* = 640) of the patients was diagnosed with a stage IV disease, compared to 12.6% (*n* = 457) in BE, 15.4% (*n* = 996) in NL, 16.7% (*n* = 698) in SE and 17.0% (*n* = 497) of the patients in NO.

**Table 1:** Patient and tumour characteristics according to country (2001–2010)

Characteristics	Belgium (*n* = 3627)^a^	Denmark (*n* = 2444)	Netherlands (*n* = 6465)	Norway (*n* = 2925)	Sweden (*n* = 4173)
*Rectal cancer*					
Sex					
Male	1766 (48.7)	1206 (49.4)	3022 (46.7)	1427 (48.8)	2175 (52.1)
Female	1861 (51.3)	1238 (50.6)	3443 (53.3)	1498 (51.2)	1998 (47.9)
Age (years)					
80–84	2213 (61.0)	1368 (56.0)	3843 (59.4)	1645 (56.2)	2421 (58.0)
85–89	1073 (29.6)	788 (32.2)	1982 (30.7)	919 (31.4)	1312 (31.4)
90–94	278 (7.7)	249 (10.2)	559 (8.6)	302 (10.3)	372 (8.9)
95–99	58 (1.6)	38 (1.6)	77 (1.2)	51 (1.7)	64 (1.5)
100+	5 (0.1)	1 (0.04)	4 (0.1)	8 (0.3)	4 (0.1)
Stage					
I	631 (17.4)	375 (15.3)	1474 (22.8)	951 (32.5)	732 (17.5)
II	858 (23.7)	550 (22.5)	1618 (25.0)	499 (17.1)	879 (21.1)
III	918 (25.3)	421 (17.2)	1331 (20.6)	539 (18.4)	887 (21.3)
IV	457 (12.6)	640 (26.2)	996 (15.4)	497 (17.0)	698 (16.7)
Unknown	763 (21.0)	458 (18.7)	1046 (16.2)	439 (15.0)	977 (23.4)
Grade					
I	562 (15.5)	^b^	310 (4.8)	^b^	128 (3.1)
II	1780 (49.1)	^b^	3026 (46.8)	^b^	1005 (24.1)
III	434 (12.0)	^b^	647 (10.0)	^b^	159 (3.8)
Unknown	851 (23.5)	^b^	2482 (38.4)	^b^	2881 (69.0)

Data are presented as *n*(%)

^a^Only data of the period 2004–2010 were available

^b^Not registered in data set

Median follow-up was 2.5 years (range: 0.0–13.5 years). Median follow-up of patients alive at the end of follow-up was 3.3 years (range: 0.0–13.5 years).

### Stage I–III rectal cancer

#### Comparison of treatment and absolute survival between neighbouring countries

**Fig. 1:**
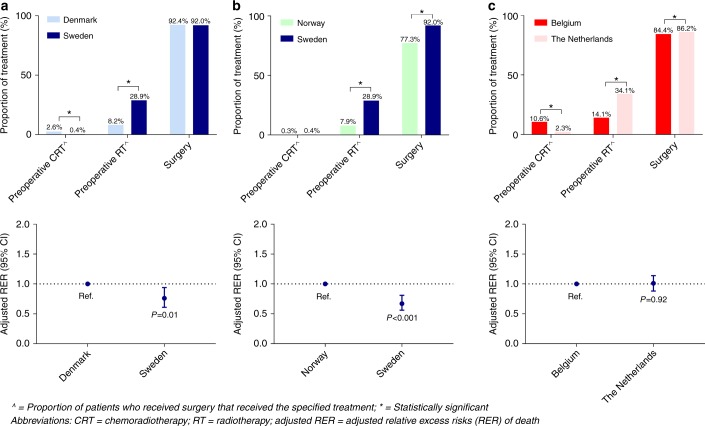
Comparison between **a** DK-SE, **b** NO-SE and **c** BE-NL regarding proportion of treatment (preoperative CRT, preoperative RT and surgery) and adjusted RER of patients with rectal cancer aged 80 years and older with stage I–III disease (2001–2010)

Stage I–III patients in SE received 29.3% preoperative chemoradiotherapy or preoperative radiotherapy in comparison with 10.8% of the Danish patients and 8.2% of the Norwegian patients (Fig. 1). More stage I–III Danish patients underwent surgery compared to Swedish patients and Norwegian patients (92.4% vs. 92.0% and 77.3%). Stage I–III Swedish patients had a significant better survival than Danish and Norwegian patients (adjusted RER 0.76, 95% CI: 0.61–0.94, *P* = 0.01; adjusted RER 0.67, 95% CI: 0.56–0.81, *P* < 0.001).

Preoperative treatment (chemoradiotherapy or radiotherapy) was given more often in NL compared to BE (36.4% vs. 24.7%, Fig. 1), whereas Belgian patients received preoperative chemoradiotherapy more often (10.6% vs. 2.3%). In NL, 34.1% of the patients received preoperative radiotherapy compared to 14.1% in BE. The rate of patients undergoing surgery in NL and BE was comparable (86.2% vs. 84.4%). Survival of Dutch patients did not differ compared to Belgian patients (adjusted RER 1.01, 95% CI: 0.88–1.14, *P* = 0.92).

#### Relative survival

RS according to country is shown in Fig. 3a. Five-year RS of stage I–III patients in SE was 72.3% (95% CI: 68.4–76.2), whereas 5-year RS in BE was 61.7% (95% CI: 58.0–65.4).

### Stage IV rectal cancer

#### Comparison of treatment and absolute survival between neighbouring countries

**Fig. 2:**
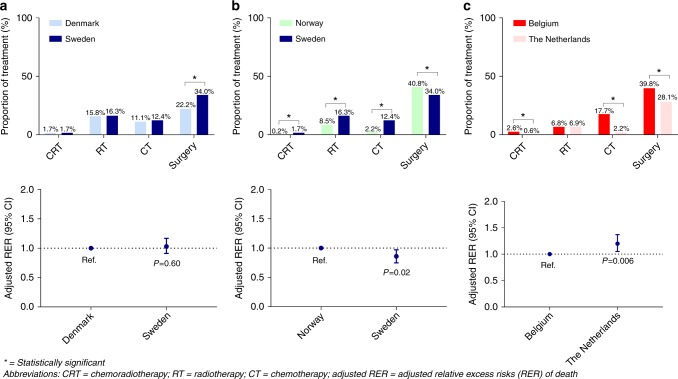
Comparison between **a** DK-SE, **b** NO-SE and **c** BE-NL regarding proportion of treatment (CRT, RT, CT and surgery) and adjusted RER of patients with rectal cancer aged 80 years and older with stage IV disease (2001–2010)

The proportion of stage IV patients in DK and SE who received chemoradiotherapy or radiotherapy (17.5% and 18.0%, Fig. 2) was higher compared to NO (8.7%). Less Norwegian patients received chemotherapy (2.2%) as compared to the Danish patients (11.1%) and Swedish patients (12.4%). Less Danish patients underwent surgery (22.2%), in comparison with 34.0% in SE and 40.6% in NO. Stage IV patients in SE had an improved survival compared to NO (adjusted RER 0.86, 95% CI: 0.75–0.97, *P* = 0.002), whereas no survival difference was observed between SE and DK (adjusted RER 1.03, 95% CI: 0.91–1.17, *P* = 0.60).

The proportions of preoperative chemoradiotherapy and radiotherapy given to stage IV patients in BE and in NL were not significantly different (9.4% vs. 7.5%). More often stage IV patients in BE (17.7%) received chemotherapy, compared to 2.2% in NL (Fig. 2). A larger proportion of the Belgian stage IV patients underwent surgery compared to the Dutch patients (39.8% vs. 28.1%). Stage IV patients in NL had an impaired survival compared to BE (adjusted RER 1.20, 95% CI: 1.05–1.37, *P* = 0.006).

#### Relative survival

**Fig. 3:**
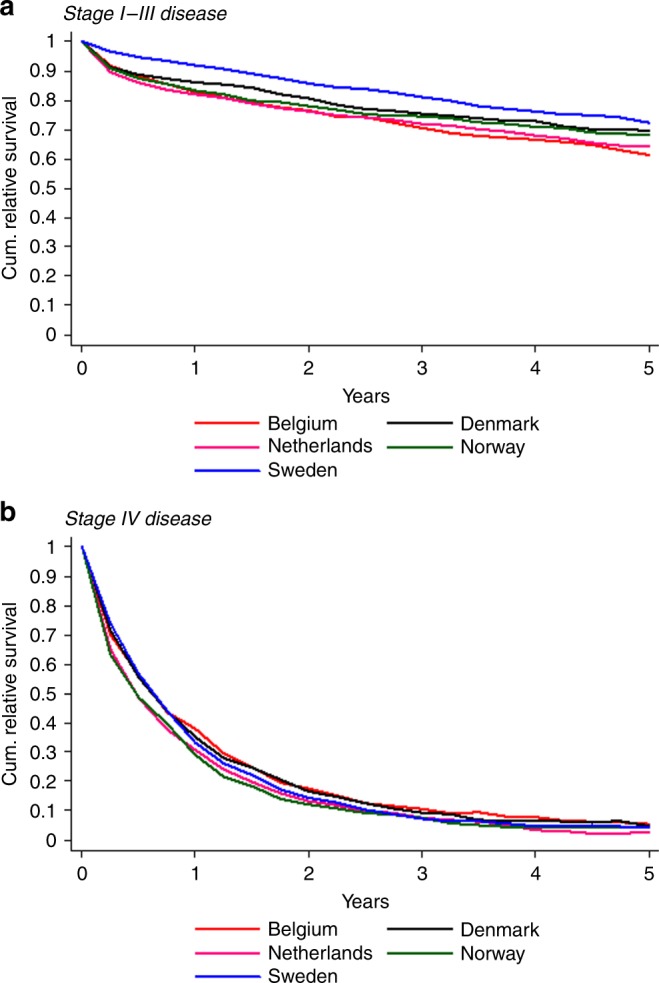
Relative survival of rectal cancer patients aged 80 years and older during 2001–2010 according to country, stratified by stage I–III (**a**)/ IV disease (**b**)

RS according to country is shown in Fig. 3b. For stage IV patients, 5-year RS in NL was 2.8 (95% CI: 1.2–5.6) compared to 5.6% in BE (95% CI: 3.0–9.5).

## Discussion

In this study, the variety of treatment strategies and survival of rectal cancer patients of 80 years or older was evaluated in a large population-based cohort from five European countries. A wide range of variation in treatment was observed, especially in the use of preoperative radiotherapy in stage I–III patients and the rate of undergoing surgery in stage IV patients. Furthermore, substantial variety in 5-year RS between countries was found.

### Stage I–III rectal cancer patients

It has been shown that that preoperative radiotherapy and TME reduces the rate of local recurrence compared to TME alone, and preoperative radiotherapy or chemoradiotherapy has played an important role in rectal cancer treatment since.^20^ However, different neoadjuvant strategies for rectal cancer care are implemented across Europe.^21^ Recently, Glimelius et al.^22^ compared local recurrence rates and survival in rectal cancer patients between NO and SE. Entirely different neoadjuvant approaches were observed; in SE, 49% of all rectal cancer patients received radiotherapy (mostly short course) compared to NO where 26% of patients received radiotherapy (mostly chemoradiotherapy). Interestingly, similar survival and in later years similar local recurrence rates were found in the two countries.^22^ In accordance with these results, the current study of elderly rectal cancer patients showed a large range of variation in the use of preoperative radiotherapy and chemoradiotherapy across the five European countries. A high proportion of the Swedish patients (29%) received preoperative radiotherapy compared to neighbouring countries NO (8%) and DK (8%). Similar rates of Swedish and Danish underwent surgery (both 92%), whereas this number was lower in NO (77%). These high percentages of operated patients in SE and DK might be a reflection of a better performance status of these patients, but this information was unfortunately not available. This could have contributed to the high 5-year survival in SE. Furthermore, this high survival curve in SE might be explained by the aggressive treatment strategy, consisting of a high proportion of patients undergoing preoperative radiotherapy and surgery.

Although it is shown that preoperative radiotherapy decreased local recurrence rate in rectal cancer patients aged 70+ compared to no or postoperative radiotherapy, a lower rate of the use of radiotherapy alone or in combination with surgery is seen compared to younger patients.^23,24^ A recent registry study of patients with rectal cancer stage I–III has shown that preoperative radiotherapy or chemoradiotherapy is associated with reduced risk of local recurrence, and tendency of improved survival, significant in patients >70 years.^25^ An explanation for the lower use of radiotherapy might be that a higher risk of recurrence may be deemed acceptable in elderly patients, as in this group maintaining health and function is of great importance in order to maintain ability of self-care.^26^ On the other hand, radiation therapy alone, for instance in combination with endorectal brachytherapy, might be an option for achieving local control, as recently explored in the HERBERT study in elderly or inoperable rectal cancer patients.^27^ A high overall response rate was observed, however, with a high rate of severe late toxicity.

Variety was seen regarding the rate of operated patients in stage I–III rectal cancer patients. In SE, 92% of the patients underwent surgery, while only 77% of the patients in neighbouring NO were operated. Several studies have shown that older rectal cancer patients are less likely to undergo surgery.^28^ In the Surveillance, Epidemiology, and End Results database between 1998 and 2009, approximately 80% of the rectal cancer patients aged <80 underwent surgery, compared to 70% of the patients between 80 and 89 years, and only 50% of the patients 90 years or older.^28^ The operated patients aged 80+ had a better survival compared to the not-operated patients, suggesting that surgery should be considered for each patient, irrespective of age.^28^ Nevertheless, due to the retrospective design of this study, selection bias is highly expected, as only a proportion of older patients underwent surgery, which may reflect a selection of patients who are physically more fit and have a better performance status.

Regarding the outcomes after surgery for older rectal cancer patients, some studies showed comparable outcomes after surgery in rectal cancer patients aged 70+, whereas other studies showed a higher rate of complications and worse survival.^3,29^ Especially for elderly, local procedures such as transanal endoscopic microsurgery should be considered as an option in order to avoid major surgery.^30^ This surgical approach is a local excision technique, suitable for well-selected T1 rectal cancer or patients with T2 rectal cancer who are unsuitable for major surgery due to comorbidity. Another suitable alternative for rectal cancer surgery might be the “watch and wait” strategy for tumours with complete response after radiotherapy.^31–33^

As in the older rectal cancer patients prognosis and treatment decisions are greatly influenced by comorbidity and frailty, a geriatric assessment has become an important component (in the preoperative phase) in the treatment of older colorectal patients.^26,34^ The International Society for Geriatric Oncology recommends that a geriatric assessment should be implemented in current guidelines in order to optimise clinical decision-making for older rectal cancer patients, so age itself should not prevent patients from receiving treatment recommended in guidelines for colorectal cancer.^26^

### Stage IV rectal cancer patients

The typical treatment backbone of stage IV rectal cancer patients comprises chemotherapy.^35^ Older rectal cancer patients have been highly underrepresented in most chemotherapy trials, although during the latest years more data have become available for this group of patients.^26^ Fit older patients seem to derive a similar benefit of combination chemotherapy (and bevacizumab), but data concerning improved survival and acceptable quality of life are still lacking for this population. Also, older rectal cancer patients are less likely to undergo radical resection compared to younger counterparts and a bigger proportion of patients receive palliative radiotherapy.

In the current study, stage IV patients in NO were approximately twice as likely to undergo surgery compared to DK (40.8% vs. 22.2%), illustrating the different treatment approaches. Currently, there is very low-quality evidence available regarding the benefit of surgical resection of the primary tumour in stage IV colorectal cancer. Some studies showed survival benefit in stage IV colorectal patients in favour of the resection group compared to the non-resection group, while other studies did not report any significant difference in survival.^36,37^

### Strengths and limitations

To our knowledge, an international comparison between European countries of rectal cancer patients aged 80+ regarding treatment and RS has not been performed before. Furthermore, the data of five different countries and the large number of patients from the national cohorts strengthen the results of this study. Considering that outcomes of older rectal cancer patients are rarely reported, outcomes of this study are valuable to determine the most optimal treatment for this population. Our study showed that substantial variation in treatment between European countries exists, emphasising the need for uniform definitions and registration of data to study outcomes of treatment strategies.^38^

Although adjusting for sex, stage and age in current analyses, residual confounding cannot be excluded. Additional confounding factors, as comorbidity and emergency surgery, were not available in the national data sets. As this study contains data of several national registries, there could be differences between these registrations such as the reliability of the data, which may have obscured the results of the current study. Data on chemotherapy in NO are for instance based on the planned treatment, which might be different from the actual received chemotherapy. Furthermore, the non-staged patients could have influenced the results as these patients were excluded for the stratified analyses. This group could contain patients who are not deemed fit for surgery due to frailty and comorbidity and as a consequence, better results might have been observed in the current study. Finally, no data were available about the chemotherapy regimens and number of courses, although recently it has been shown that chemotherapy is increasingly used in older stage IV colorectal patients.^39^

## Conclusion

In conclusion, this observational international comparison across five countries of rectal cancer patients aged 80+ showed a wide range of variation in treatment strategy, especially in the use of preoperative radiotherapy in stage I–III patients and the rate of undergoing surgery in stage IV patients. Moreover, variations in 5-year RS in stage I–III patients were observed. A clear pattern between treatment and survival was not observed. Further research into selection criteria for certain treatment strategies could lead to tailored treatment for older rectal cancer patients in order to achieve the ultimate aim of improving outcomes in this growing group of patients.
